# THE ROLE OF COLLECTIVISM IN EXPLAINING LIFE COURSE HEALTH DISPARITIES IN OLDER AGES

**DOI:** 10.1093/geroni/igz038.1820

**Published:** 2019-11-08

**Authors:** Joelle Saad-Lessler, Karen Richman

**Affiliations:** 1 Stevens Institute of Technology, School of Business, Hoboken, New Jersey, United States; 2 University of Notre Dame, Notre Dame, Indiana, United States

## Abstract

Collectivism refers to the social practice of investing in and relying on one’s social network, rather than formal institutions, to ensure personal security. Using the re-engineered 2014 Survey of Income and Pension Participation (SIPP), we investigate how collectivist practices affect life course health disparities at older ages in the US. Indicators of Collectivism include measures of caregiving, inter and intrahousehold financial and material support and help from charities, friends and family members. Regression results indicate that increased collectivist interactions are associated with improved self-reported health status outcomes. Government support for collectivist behaviors can thus yield a low cost means of improving health outcomes among the elderly.

